# Evolution of Drug-resistant *Acinetobacter baumannii* After DCD Renal Transplantation

**DOI:** 10.1038/s41598-017-01683-7

**Published:** 2017-05-16

**Authors:** Hong Jiang, Luxi Cao, Lihui Qu, Tingting Qu, Guangjun Liu, Rending Wang, Bingjue Li, Yuchen Wang, Chaoqun Ying, Miao Chen, Yingying Lu, Shi Feng, Yonghong Xiao, Junwen Wang, Jianyong Wu, Jianghua Chen

**Affiliations:** 1 Kidney Disease Center, the First Affiliated Hospital, College of Medicine, Zhejiang University, Hangzhou, 310003 P. R. China; 2 Kidney Disease Immunology Laboratory, the Third Grade Laboratory, State Administration of Traditional Chinese Medicine, Hangzhou, P. R. China; 3 Key Laboratory of Multiple Organ Transplantation, Ministry Of Health, Hangzhou, P. R. China; 4 Key Laboratory of Nephropathy, Zhejiang Province, Hangzhou, P. R. China; 5 State Key Laboratory for Diagnosis and Treatment of Infectious Disease, First Affiliated Hospital, School of Medicine, Zhejiang University, Hangzhou, 310003 Zhejiang China; 6 Collaborative Innovation Center for Diagnosis and Treatment of Infectious Diseases, Hangzhou, China; 7 Department of Health Sciences Research and Center for Individualized Medicine, Mayo Clinic, Scottsdale, AZ 85259 USA; 8 Department of Biomedical Informatics, Arizona State University, Scottsdale, AZ 85259 USA

## Abstract

Infection after renal transplantation remains a major cause of morbidity and death, especially infection from the extensively drug-resistant bacteria, *A*. *baumannii*. A total of fourteen *A*. *baumannii* isolates were isolated from the donors’ preserved fluid from DCD (donation after cardiac death) renal transplantation and four isolates in the recipients’ draining liquid at the Kidney Disease Center, The First Affiliated Hospital, College of Medicine, Zhejiang University, from March 2013 to November 2014. An outbreak of *A*. *baumannii* emerging after DCD renal transplantation was tracked to understand the transmission of the pathogen. PFGE displayed similar DNA patterns between isolates from the same hospital. Antimicrobial susceptibility tests against thirteen antimicrobial agents were determined using the K-B diffusion method and eTest. Whole-genome sequencing was applied to investigate the genetic relationship of the isolates. With the clinical data and research results, we concluded that the *A*. *baumannii* isolates 3R1 and 3R2 was probably transmitted from the donor who acquired the bacteria during his stay in the ICU, while isolate 4R1 was transmitted from 3R1 and 3R2 via medical manipulation. This study demonstrated the value of integration of clinical profiles with molecular methods in outbreak investigation and their importance in controlling infection and preventing serious complications after DCD transplantation.

## Introduction

The Minister of Health of the People’s Republic of China has announced that the Chinese government will comply with international ethical standards and abolish organ harvesting from executed prisoners^1^. The human organ donation system was established in China in August 2009 ^2^. At the beginning of 2010, on an experimental basis, human organ donation was first performed in Tianjin, Shanghai, Liaoning, Hunan, Zhejiang and six other provinces under the instruction of the China Red Cross Association and the Ministry of Health^3, 4^. The whole donation system was not implemented nationwide until March 2012. However, the Chinese government has proclaimed to stop organ harvesting from executed prisoners starting January 1, 2015^5^. Our center, as one of the first experiment institutions, has completed 680 cases of donation after cardiac death (DCD), accumulated by January 2016, thus ranking among the top in national list of DCD.

DCD has become an important source of donor organs in China. Patients in intensive care units (ICUs) are the main source of DCD donors because they are well supported by life support equipment; thus, the medical group can take timely action, and the organ procurement team can remove the equipment in an organized way with the consent of family members. With treatments such as tracheal intubation, deep vein catheterization and the use of other life support equipment, these patients are susceptible to serious bacterial infection. Therefore high-dose antibiotics will be used for the patients for a long time, leading to a high risk of developing nosocomial multiple drug-resistant (MDR) bacterial infections and extensive drug-resistant (XDR) bacterial infections.

With the improvement in surgical techniques and the development of novel immunosuppressants, the survival rate of patients receiving kidney transplantation has increased, and these patients have experienced life quality improvement^6^. However, early infection after transplantation is still not well controlled, especially for MDR and XDR^7–11^. For example, patients who are infected with *Acinetobacter baumannii* have higher mortality after kidney transplantation, which makes *A*. *baumannii* infection an important issue influencing long-term prognosis. Previous studies focusing on *A. baumannii* infection after DCD kidney transplantation^12–16^ were either presented in the form of a case report that occurred in a single medical center or limited in emphasizing descriptions of clinical manifestation, therapeutic regimen and prognosis of several infected cases. Though all researches and clinical phenomena noted that the source of infection was most likely from the donors, adequate and more well-grounded evidence needs to be presented. It is still a question that where the pathogens come from (the donors, the recipients, or others) and how they spread (the exact transmission route). Moreover, a scientific investigation on infection induced by *A. baumannii* after DCD kidney transplantation to map the transmission route of an outbreak is still absent. If we could integrate clinical and epidemiological profiles with molecular methods such as Pulsed-field gel electrophoresis (PFGE) or Whole Genome Sequencing (WGS), a more accurate way to control infection and prevent serious complications could be proposed in time to improve prognosis.

Effective control of *A. baumannii* outbreak requires a detailed understanding of how transmission occurs. With rapid development of technology, PFGE, multi-locus sequence typing and WGS have been applied widely in various studies involving microbiology. In particular, WGS is emerging as the gold standard in bacterial typing^17, 18^, which facilitates our understanding of the spread of infectious agents in clinically nosocomial strains, using a limited study size or scope in reconstructing transmission links during the course of the outbreak^19–23^.

In this study, PFGE and WGS were used to track the spread of *A. baumannii* after DCD renal transplantation. Additionally, we displayed epidemiological clues (patient location and overlap during the transmission, putative map of transmission, patient trace) for infection control procedures that are intended to prevent serious complications.

## Materials and Methods

### Isolates and antimicrobial susceptibility testing

A total of eighteen *A. baumannii* isolates, which are stored in our biobank, were isolated from fourteen preservation fluid samples of nine donors and four draining liquid samples of three recipients, when they underwent DCD renal transplantation at the Kidney Disease Center, the First Affiliated Hospital, College of Medicine, Zhejiang University, from March 2013 to November 2014. Kidney preservation fluid was collected for routine cultures before surgery. Draining liquid was collected from a drainage tube routinely placed for blood oozing and fluid oozing after transplant surgery. After surgery we routinely collect recipients’ drainage liquid for culture at least for 3 times. We named the isolates on basis of its source of specimen. D stands for donor and R stands for recipient. There is one-to-one correspondence between the order of donor and recipient. For example, 3R1 was from the draining liquid of the recipient who received the kidney of 3D1, and 3R2 was isolated from the draining liquid of the recipient who received the other kidney of 3D2. Isolate 3D1 and 3D2 were cultured separately from the kidney preservation fluid of donor 3. The antimicrobial susceptibilities of all of the strains against 13 antimicrobial agents were determined by the disk diffusion method based on the standard Kirby–Bauer method (gentamicin, amikacin, piperacillin, piperacillin/tazobactam, ceftazidime, cefotaxime, cefepime, imipenem, meropenem, ciprofloxacin, aztreonam) and E-test (tigecycline and polymyxin) in accordance with the 2016 guidelines of the Clinical and Laboratory Standards Institute (CLSI)^24^. The results gained by K-B method were interpreted in accordance with the 2016 guidelines of CLSI^24^. The value of minimum inhibitation concentration [MIC (ug/mL)] obtained by E-test were interpreted based on EUCAST standard^25^.

### Pulsed-field gel electrophoresis (PFGE)

All the eighteen *A. baumannii* isolates were classified according to PFGE analysis. After the genomic DNA was digested with *Apa*I (Sangon, Shanghai, China), the DNA fragments were separated by electrophoresis in 1% agarose III (Sangon) in 0.5× Tris-borate-EDTA buffer with a CHEF apparatus (CHEF Mapper XA, Bio-Rad, USA). The conditions included 14 °C and 6 V/cm with alternating pulses at a 120° angle with a 5–35 s pulse time gradient for 22 h. *Salmonella enterica* serotype Braenderup H9812 was used as the size marker^26^. The similarity of DNA patterns were analyzed with BioNumerics 7.0 (Applied Math, USA). DNA patterns differing by ≥1 PFGE bands were considered distinct.

### Whole-genome sequencing

The total DNA was extracted from the eighteen *A. baumannii* isolates and sequenced using next-generation sequencing technology (Illumina HiSeq2500^TM^ with 2 × 125 bp paired-end reads). The derived short reads were assembled into contigs using CLC Genomics Workbench 8.0.1 (CLCbio, Denmark). MLSTs were identified by mapping the assembled contigs against the *A. baumannii* MLST database on the CGE server. A threshold of 2,500 SNVs was considered accurately distinguished *A. baumannii* isolates from different clonal lineages^27^.

## Results

### The clinical characteristics of 18 *A. baumannii* isolates

A total of eighteen *A. baumannii* isolates were stored in the biobank after being isolated from 14 preservation fluid samples of 9 donors and 4 draining liquid samples of 3 recipients who underwent DCD renal transplantation at the Kidney Disease Center, the First Affiliated Hospital, College of Medicine, Zhejiang University, from March 2013 to November 2014. Among these isolates, 1D1 and 1D2 were from preservation fluids of kidneys of the same donor, so were 3D1 and 3D2, 6D1 and 6D2, 7D1 and 7D2, 9D1 and 9D2. 4R1 and 4R1b were isolated from the draining liquid at different time points of the same recipient, whose donor kidney was 4D1. However, the preserved fluid of 4D1 was not infected by *A. baumannii*, while 4D2 and 4R2 were not infected by *A. baumannii*. In addition, 10D1 was infected by *A. baumannii*, but none of 10R1, 10D2, and 10R2 was infected by *A. baumannii*. The detailed clinical characteristics are shown in Table 1.

**Table 1:** Clinical information of matched donors and recipients.

Donor	Recipient	Recipient Age	Recipient Gender	Donor Hospital	Isolate type	Isolated date	Recipient Outcome
1D1	1R1	50	M	Ho1	preservation fluid	2013-12-05	no sign of clinical infection
1D2	1R2	50	F	Ho1	preservation fluid	2013-12-05	no sign of clinical infection
2D1	2R1	45	F	Ho2	preservation fluid	2014-05-19	no sign of clinical infection
3D1	3R1	62	M	Ho3	preservation fluid	2014-08-30	recover
					draining liquid	2014-08-31	
3D2	3R2	49	F	Ho3	preservation fluid	2014-08-30	recover
					draining liquid	2014-08-31	
4D1	4R1/4R1b	39	M	Ho4	draining liquid	2014-09-15	recover
					draining liquid	2014-09-29	
5D1	5R1	61	M	Ho5	preservation fluid	2014-03-31	no sign of clinical infection
6D1	6R1	44	F	Ho5	preservation fluid	2013-09-11	no sign of clinical infection
6D2	6R2	46	M	Ho5	preservation fluid	2013-09-11	no sign of clinical infection
7D1	7R1	57	M	Ho7	preservation fluid	2014-04-16	no sign of clinical infection
7D2	7R2	38	M	Ho7	preservation fluid	2014-04-16	no sign of clinical infection
8D1	8R1	58	M	Ho8	preservation fluid	2013-08-11	no sign of clinical infection
8D2	8R2	44	F	Ho8	preservation fluid	2013-08-11	no sign of clinical infection
9D1	9R1	45	F	Ho9	preservation fluid	2013-04-03	no sign of clinical infection
10D1	10R1	59	F	Ho10	preservation fluid	2014-08-08	no sign of clinical infection

### Pulsed-field gel electrophoresis (PFGE)

**Figure 1:**
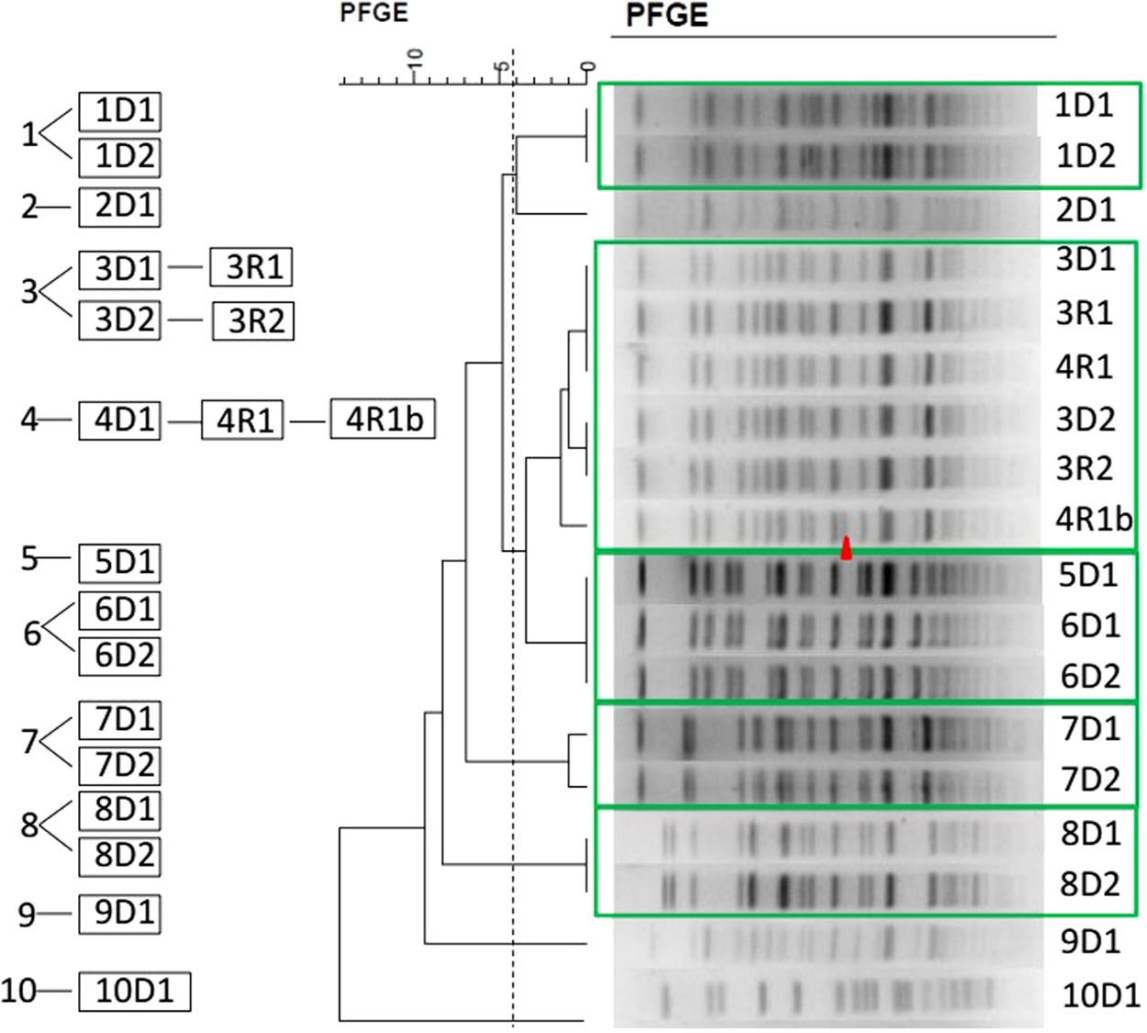
PFGE profiles of ApaI-digested DNA (all of the DNA) from 18 *A*. *baumannii* isolates. PFGE bands of the 18 *A. baumannii* isolates.

The similarities of the PFGE electrophoresis bands between 1D1 and 1D2, 6D1 and 6D2, 7D1 and 7D2, 9D1 and 9D2 analyzed by BioNumerics were up to 95% (Fig. 1), since each pair of kidneys came from one donor. 5D1 displayed similar PFGE electrophoresis bands to that of 6D1 and 6D2, with the homology up to 95%, which is consistent with the fact that the two donors came from the same ICU of hospital 5 (as shown in Table 1). The 5 *A. baumannii* isolates from 3D1, 3R1, 3D2, 3R2, and 4R1 displayed the same PFGE electrophoresis bands except for one band in 4R1b. It can be deduced that 3R1 and 3R2 were transmitted from 3D1 and 3D2, whereas 4R1 and 4R1b were not from 4D1, since the preserved fluids of 4D1 was free of *A. baumannii*. The slight difference between 4R1 and 4R1b suggested a dynamic change in DNA sequences. For this reason, it was necessary to perform a WGS for a higher resolution comparison.

### Whole-genome sequencing

**Figure 2:**
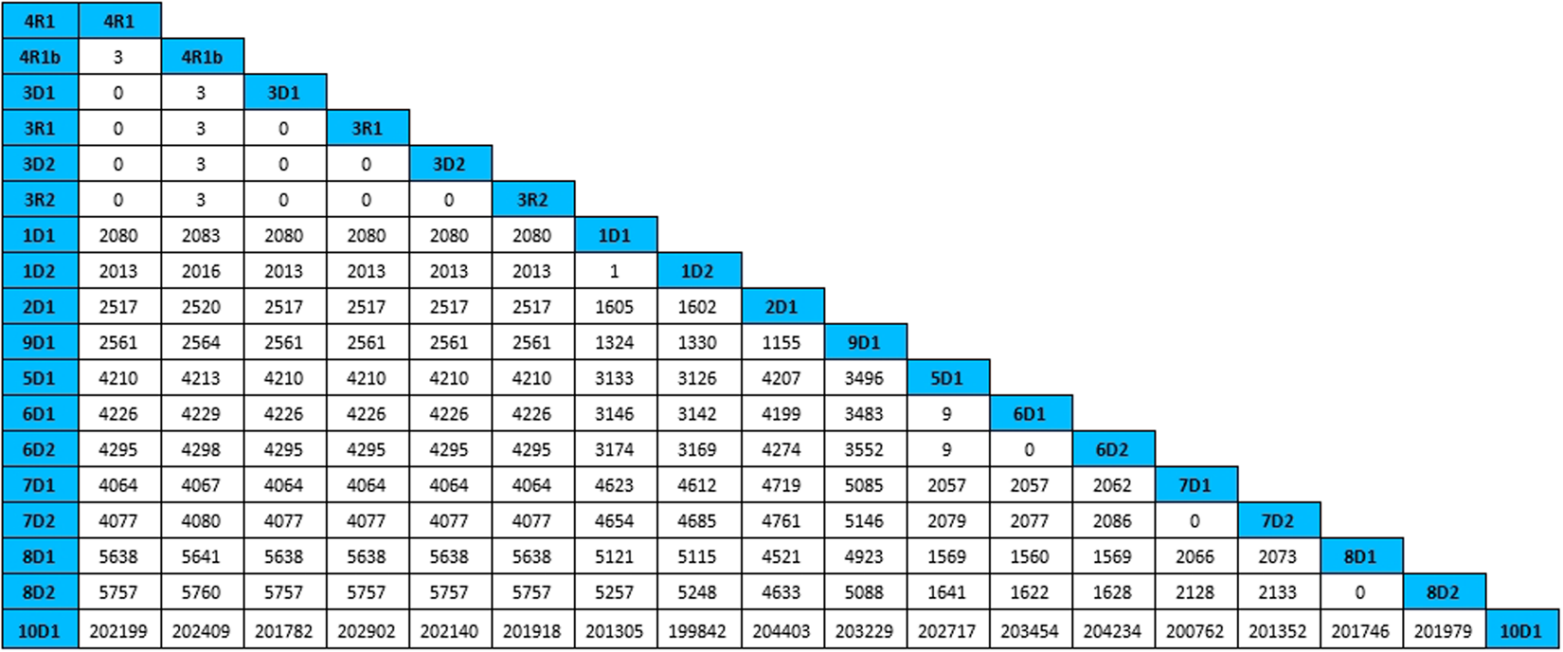
Mutations identified by genome comparison between every two *A.baumannii* isolates. Isolate 10D1 has a much larger genome than that of the other isolates, so are the numbers of SNVs between it and other isolates. Result of BLAST suggested that it was identical to the type genome of *Acinetobacter nosocomialis*.

**Figure 3:**
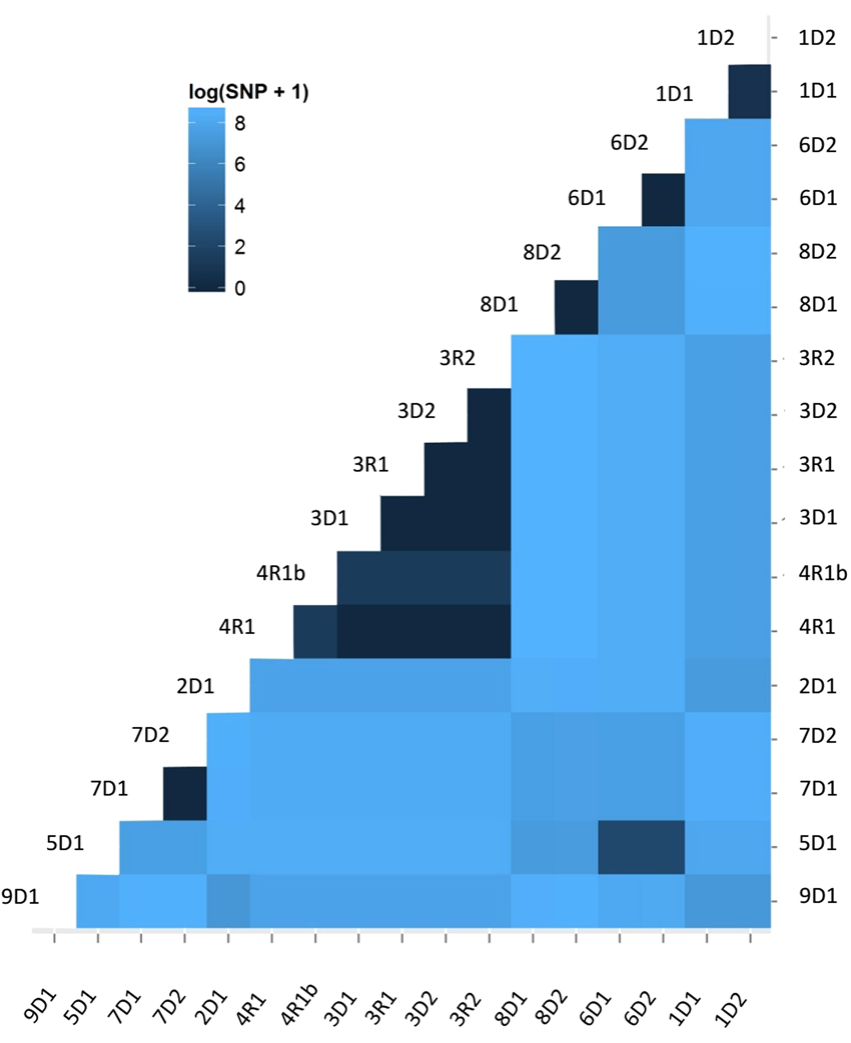
Heatmap. SNVs among all of the outbreak genomes are shown in a clustered heat map that is calculated by the log value (+1, in the case of an occurrence of zero) of the DNA sequence variations among the isolates. The shade degree of each grid represents the SNVs between two genomes. The deeper the color is, the more similar the genome.

Table 2 and Fig. 2 show the Single Nucleotide Variants (SNVs) between each pair of isolates revealed by whole-genome sequencing comparison. There was 1 SNV between 1D1 and 1D2, 0 SNVs between 7D1 and 7D2, and 0 SNVs between 8D1 and 8D2. There were 0 SNVs between 6D1 and 6D2 and 9 SNVs between 5D1 and 6D1&2. These pairs of isolates are almost identical to each other, so were 3D1, 3D2, 3R1, 3R2, and 4R1. There were 3 SNVs between 4R1b and 3D1, 3D2, 3R1, 3R2, 4R1. 4R1b was a heteromorphosis isolate that varied from the 4R1 isolate. Previous studies indicated that common mutant position inducing tigecycline-resistance may be located in the AdeS gene which regulates the expression of efflux pump^28–31^. The sequence size of the isolate from 10D1 has much larger genome than the other isolates, leading to large number of SNVs different from other isolates. Furthermore, BLAST result suggested that isolate 10D1 was identical to the genome of *Acinetobacter nosocomialis*, which belongs to the *Acinetobacter calcoaceticus-baumannii* complex. For this reason, this isolate was not shown on the heatmap. In the heatmap (Fig. 3), the shade degree of each grid represents the number of SNVs between two genomes. The deeper the color is, the more similar the genomes are. The WGS data of the isolates were deposited to NCBI genebank (Bioproject: PRJNA322764).

**Table 2:** Antibiotic susceptibility profiles of the 18 clinical isolates of *A*. *baumannii* [Zone diameter (mm) and MIC (ug/mL)].

Donors	1D1	1D2	2D1	3D1		3D2		4D1		5D1	6D1	6D2	7D1	7D2	8D1	8D2	9D1	10D1
Recipients	1R1	1R2	2R1	3R1		3R2		4R1	4R1b	5R1	6R1	6R2	7R1	7R2	8R1	8R2	9R1	10R1
Isolate type	PF	PF	PF	PF	DL	PF	DL	DL	DL	PF	PF	PF	PF	PF	PF	PF	PF	PF
**Zone diameter (mm)**																		
PP	6	6	6	6	6	6	6	6	6	6	6	6	6	6	6	6	6	19
PTC	6	6	6	6	6	6	6	6	6	6	6	6	6	6	6	6	6	22
TZ	6	6	6	6	6	6	6	6	6	6	6	6	6	6	6	6	6	19
CT	6	6	6	6	6	6	6	6	6	6	6	6	6	6	6	6	6	17
PM	6	11	6	6	6	6	6	6	6	6	6	6	10	9	6	6	6	20
IP	6	6	6	6	6	6	6	6	6	6	6	6	6	6	6	6	6	26
MP	6	6	6	6	6	6	6	6	6	6	6	6	6	6	6	6	6	24
AT	6	6	6	6	6	6	6	6	6	6	6	6	10	6	6	6	6	13
GM	6	6	6	6	6	6	6	6	6	6	6	6	6	6	6	6	6	19
AK	6	6	6	6	6	6	6	6	6	6	6	6	6	6	6	6	18	19
CI	6	6	6	6	6	6	6	6	6	6	6	6	6	6	6	6	6	22
**MIC (mg/L)**																		
TGC	1.5	3	1.5	2	3	3	2	2	8	3	3	3	3	3	2	2	2	0.125
PO	0.5	0.5	0.5	0.5	3	0.5	0.5	0.5	0.5	0.5	0.5	0.5	0.5	0.5	0.5	0.5	0.38	0.5

^※^PP: Piperacillin, PTC: Piperacillin/Tazobactam, TZ: Ceftazidime, CT: Cefotaxime, PM: Cefepime, IP: Imipenem, MP: Meropenem, AT: Aztreonam, GM: Gentamicin, AK: Amikacin, CI: Ciprofloxacin, TGC: Tigecycline, PO: Polymyxin.

^※^PF: preservation fluid, DL: draining liquid.

### Antimicrobial susceptibility testing

The antimicrobial susceptibilities of all isolates against thirteen antimicrobial agents were determined by the disk diffusion method based on the standard Kirby–Bauer method (gentamicin, amikacin, piperacillin, piperacillin/tazobactam, ceftazidime, cefotaxime, cefepime, imipenem, meropenem, ciprofloxacin, aztreonam) and E-test (tigecycline and polymyxin). The results gained by K-B method were interpreted in accordance with the 2016 guidelines of the Clinical and Laboratory Standards Institute (CLSI)^24^. The value of minimum inhibition concentration [MIC (ug/mL)] obtained by E-test were interpreted based on EUCAST standard^25^. All the isolates were revealed to be XDR *A. baumannii* (resistant to all but 1 or 2 classes of antimicrobial, mainly referring to tigecycline and colistin^32^) except strain 9D1 and 10D1^33^. 10D1 was shown to be a sensitive *A. baumannii* isolate. The detailed results are shown in Table 2.

### The transmission map shows the donors from the different Hospital ICUs in the different cities of Zhejiang Province

The donor of 3D1 and 3D2 was hospitalized in the ICU of Hospital 3, while the donor of 4D1 was hospitalized in the ICU of Hospital 4. Hospital 9 was the recipients’ hospital. Hospitals 7 and 9 were located in the same city (Hangzhou). Figure S1 shows the transmission map of the donors from the different Hospital ICUs in the different cities of Zhejiang Province.

### Patient trace

**Figure 4:**
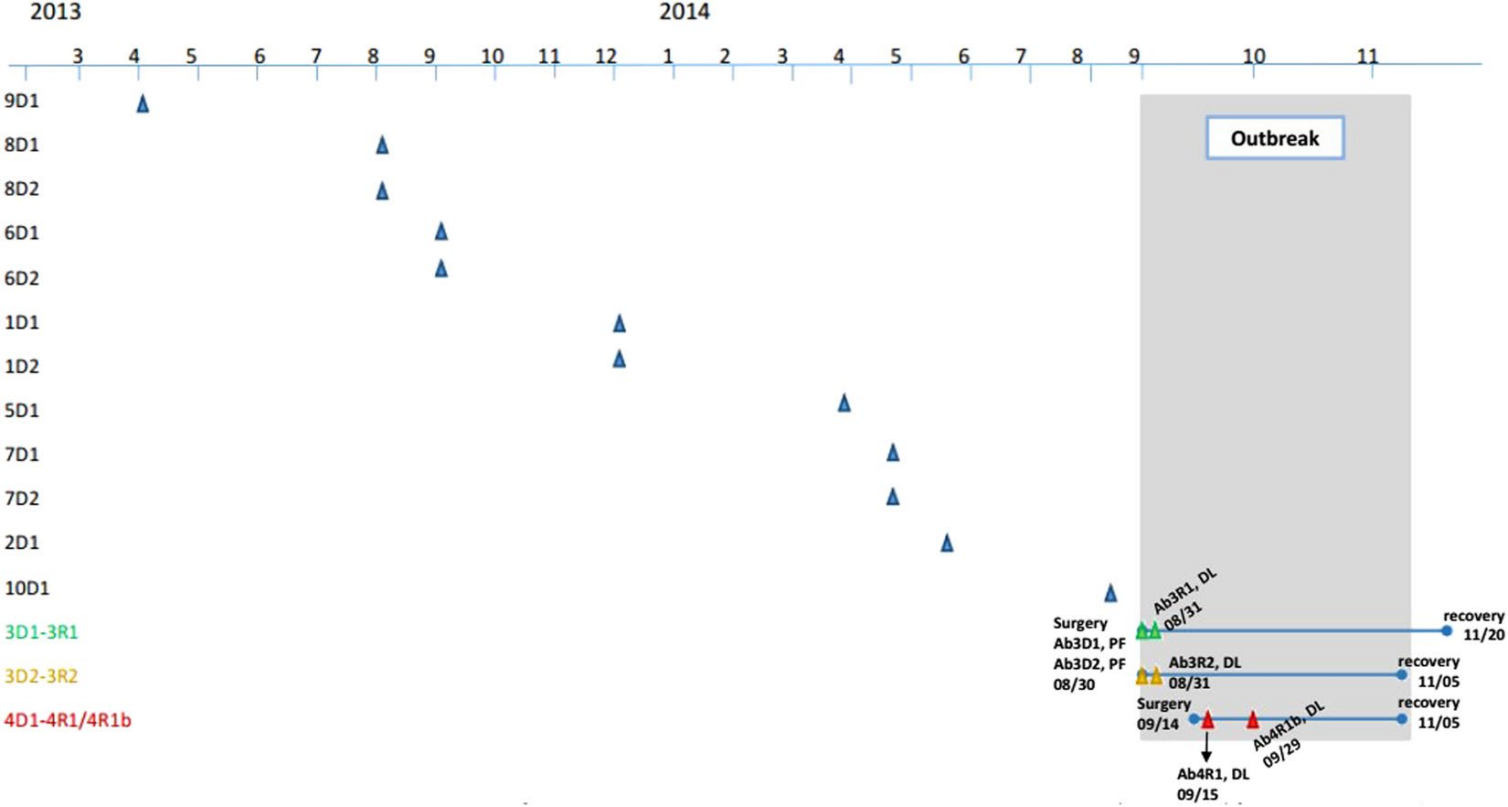
Patient trace. Timeline of first positive cultures (refering to the first time that *A*. *baumannii* was identified in the liquid samples) of the outbreak isolates. The blue triangles represent *A*. *baumannii* isolates gained from preservation fluid of donors. The green (3D1-3R1), yellow (3D2-3R2), red (4R1) triangles represent the first positive cultures of outbreak *A*. *baumannii* isolates. Dates are given as month/date. Culture of 3D1’s kidney preservation fluid sample collected on August 30th grew *A*. *baumannii*, and culture of draining liquid collected after the transplantation (August 31st) for 3R1 grew *A*. *baumannii* as well. The same situation occurred in 3D2 and 3R2.

On April 3, 2013, we found the first *A. baumannii* infection in the kidney preservation fluid of 9D1 in Hospital 9. Subsequently, six additional *A. baumannii* infections were identified in the kidney preservation fluid of 8D1 and 8D2, 6D1 and 6D2, 1D1 and 1D2 in August, September and December of 2013 and other five were identified in the kidney preservation fluids of 5D1, 7D1 and 7D2, 2D1, 10D1 in March, April, May and August of 2014. On August 30, 2014, two *A. baumannii* isolates were identified in the preservation fluid of paired kidneys from one donor (3D1 and 3D2). Besides, the draining liquid of the two recipients were also infected by *A. baumannii*. Recipients 3R1 and 3R2 both experienced *A. baumannii* infection. In addition to positive culture results of drainage liquid, their WBC and neutrophil increased. Besides, blood culture results of recipient 3R2 were positive of *A. baumannii*. As for clinical symptoms, recipient 3R1 suffered from pancreatitis (*A*. *baumannii*) and peritonitis (*A*. *baumannii*). Recipient 3R2 had prolonged fever, pus, neoplasm (an outgrowth from the patient’s surgical incision of which bacteria culture showed positive of *A. baumannii*) and tenderness around the tube. Subsequently, *A. baumannii* was identified in the draining liquid of recipient 4R1, yet the result of the kidney preservation fluid culture of 4D1 was not positive. WBC and neutrophil of recipient 4R1 increased. He had tenderness and ecchymosis in skin around the tube. Also 4R1 experienced prolonged fever and diarrhea. 4R1b was the isolate identified to be tigecycline-resistant during recipient 4R1’s stay in hospital. Detailed information is shown in Fig. 4. After anti-infection treatment, blood test showed that WBC of the three recipients decreased. Drainage culture results were negative for at least 3 times and clinical symptoms disappear.

### Location of Patient beds in the laminar flow ward of the Kidney Disease Center

Figure S2 displays the location of patient beds where infected recipients stayed in the laminar ward of the Kidney Disease Center. Recipient 3R1 and 3R2 shared the same room, while recipient 4R1 was hospitalized three doors next to them.

### Putative map of *A. baumannii* transmission during the outbreak

Figure S3 shows the transmission map concluded on the base of PFGE, genetic and patient trace data. 3D1 and 3D2 came from the same donor, who was treated in the ICU of Hospital 3. The PFGE and genetic data both showed that the *A. baumannii* infections in the preservation fluids and draining liquids were of the same origin. The *A. baumannii* transmission should be from the preservation fluids to the draining liquids. There was no *A. baumannii* growing in the preservation fluid culture of 4D1. The *A. baumannii* isolate in the draining liquids of 4R1 was shown to be the same as that in 3D1 and 3D2 as evidenced by the genetic and antimicrobial susceptibility testing data. 4R1b was the heteromorphosis strain that varied from the 4R1 strain.

## Discussion

In this study, *A. baumannii* isolates were identified in fourteen samples of the kidney preservation fluid. Results of PFGE revealed the same DNA fingerprints of *A. baumannii* isolated from preservation fluid of paired kidneys, which was in coincidence with clinical information that the samples of preservation fluid came from eight different donors from eight hospitals in seven different cities of Zhejiang Province. We also noticed that PFGE fingerprints of the isolates from 3D1, 3D2, 3R1, 3R2, and 4R1 were the same, while 4R1b was one band distinguished from the five isolates. The results of genome comparison of the six isolates reveal that isolates from 3D1, 3D2, 3R1, 3R2, and 4R1 have identical genomes, while 4R1b was a variant isolate containing 3 SNVs. Clinical data showed that the kidneys transplanted to recipients 3R1 and 3R2 were from the same donor hospitalized in the ICU of Hospital 3 and they shared a room after the surgery, leading to the conclusion that the four isolates from 3D1, 3D2, 3R1 and 3R2 were from the same origin. Interestingly, another transplantation patient hospitalized in the same room with them was not infected by *A. baumannii*. A reasonable conclusion can be drawn that the isolate from 4R1 may be transmitted from 3R1 or 3R2 via medical manipulation (such as B-scan ultrasonography) by medical staff, for the fact that the preservation liquid of 4D1 was not infected by *A. baumannii* and recipient 4R1 was three doors away from 3D1 and 3D2. The former fact excluded transmission from the donor, while the later fact excluded the possibility of direct contact transmission. Additionally, 4R1 was the only one infected by the same *A. baumannii* isolate as those of 3R1’s and 3R2’s. No other patients developed *A. baumannii* infection during the same period in the lamina flow ward. Cultures of the draining liquid from recipients 3R1, 3R2 and 4R1 all grew *A. baumannii*. The results of PFGE, WGS and BLAST provided a reasonable explanation that 3R1 and 3R2 were from the same origin and 4R1 was probably transmitted from 3R1 or 3R2 via medical manipulation.

The *A. baumannii* isolate from 9D1 whose donor was hospitalized in Hospital 9 was phylogenetically distant from the isolates from 4R1, 3D1, 3D2, 3R1, and 3R2. Moreover, EUCAST and FDA interpretations based on the results of susceptibility test suggested that all the isolates were XDR except isolates from 9D1 and 10D1. However, *A. baumannii* isolates including 4R1b became tigecycline-resistant after the regimen containing venous tigecycline.

Bacteremia caused by *A. baumannii* in the donors was not observed. One reasonable explanation is that the amount of *A. baumannii* in the blood was not sufficient enough to cause bacteremia. When the transplanted kidneys were transported in the preservation fluid, the preservation fluid provided a suitable environment for *A. baumannii* colonization and culturing. The isolates from the preservation fluid could be considered originating from the donors. Other reasons are listed as following. Firstly, two paired recipients showed the same DNA footprint with that of the paired donor kidneys. Secondly, the donors from different hospitals displayed different DNA footprints. Thirdly, although 5D1, 6D1, and 6D2 were from different donors but their PFGE bands and genomes were identical, indicating that the three isolates were of the same origin, which was in accordance with the fact that they came from the same ICU. If there was a small chance for medical staff to contaminate the preservation fluid, the *A. baumannii* isolates obtained should be occasional and random. Isolate from 10D1, BLASTed to be *A. nosocomialis*, is more likely to be attributed to contamination of the preservation fluid. Moreover, not all microbial contamination in the preservation fluids would give rise to an infection in recipients because such a transmission still depends on individual susceptibility and anti-infective therapy^34–36^. However, we did not acquire all the *A. baumannii* isolates from donors during that period. It is difficult to investigate further without other samples and detailed information, such as the blood of the donor or the isolates from the ICU where the donors stayed.

Together, these findings support that the *A. baumannii* isolates from 3R1 and 3R2 are probably stemmed from the donor and the ICU where the donor stayed. However, later culture of swaps from the ICU environment of Hospital 3 didn’t grow *A. baumannii* due to a long delay of sampling. In addition, the *A. baumannii* isolate from 4R1 was transmitted from 3R1 and 3R2 via medical manipulation. The six *A. baumannii* isolates (3D1, 3D2, 3R1, 3R2, 4R1 and 4R1b) were of the same origin. All the *A. baumannii* isolates studied in this research except 9D1 and 10D1 exhibited XDR phenotypes, and 4R1b turned resistant to tigecycline from a sensitive isolate after venous administration of tigecycline. This was the first time that the value of molecular methods and WGS was used in outbreak investigation of *A. baumannii* infection after DCD kidney transplantation. Although we could not determine the exact source of the outbreak strain because no positive result of environment screening was available, the evidences we obtained were sufficient to draw a reasonable transmission map of the outbreak. Infection control is crucial to limit the spread of the pathogen especially in those who are experiencing immunosuppression. Scientific outbreak investigation after DCD kidney transplantation could provide clues for infection-control procedures to prevent serious complications.

Our study has some limitations. As a retrospective study, it is difficult to trace other patients who was the carrier of the same strain. Besides, we did not obtained all specimens (blood, throat swab, etc.) of the donors during their hospitalization in the ICU. It is difficult to know about the donors’ infection status and reconstruct the distribution of microorganism in the ICU environment. We were unable to determine a primary origin of this organism since results of environmental screening were negative. There has been a long interval between sampling and the donor’s hospitalization.

In addition to close observation of organ recipients, cooperation between public health institutions should be enhanced to screen appropriate donors. Contaminated organs and their recipients must be treated with effective antibiotics based on results of antimicrobial susceptibility test to prevent further infections. Besides, we should pay more attention to application of lab techniques in preventing transmission of pathogens among patients.

## Electronic supplementary material

Figure S1-S3
