# Supplementary material for: Evolution of Drug-resistant Acinetobacter baumannii After DCD Renal Transplantation
Source: Sci Rep. 2017 May 16;7:1968. doi: 10.1038/s41598-017-01683-7 (PMC5434021; doi:10.1038/s41598-017-01683-7)
Supplement: Supplementary file 1 — Figure S1-S3 [file 41598_2017_1683_MOESM1_ESM.pdf]

Evolution of Drug-resistant *Acinetobacter baumannii* After DCD Renal Transplantation

Hong Jiang[1,*,ϕ], Luxi Cao[1,ϕ], Lihui Qu[1,ϕ], Tingting Qu[2], Guangjun Liu[1], Rending Wang[1], Bingjue Li[1], Yuchen Wang[1], Chaoqun Ying[2], Miao Chen[1], Yingying Lu[1], Shi Feng[1], Yonghong Xiao[2], Junwen Wang[3,4,*], Jianyong Wu[1,*], Jianghua Chen[1,*]

Supplementary fig 1.

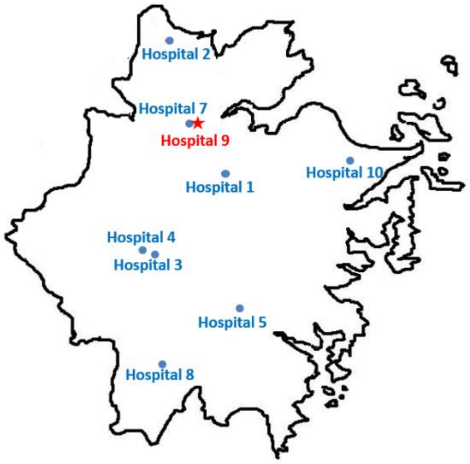

Fig S1. The transmission map of the donors from the different Hospital ICUs in different cities of Zhejiang Province. Almost all of the donors were paired with a hospital. Donor 5 and 6 came from the same hospital (Hospital 5). Hospitals 3 and 4 were in the same city. Hospital 7 and 9 were in the same city. Hospital 9 was also the recipients' hospital. This figure was drawn using Microsoft Paint (Microsoft windows version number 1607/internal version 14393.693. https://www.microsoft.com/en-us/legal/intellectualproperty/copyright/default.aspx. 12/02/2017.).

Supplementary fig 2.

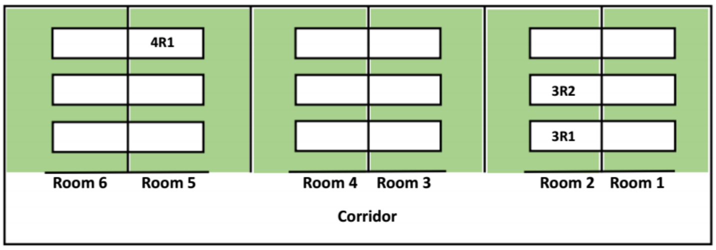

Fig S2. Unit floor plans. Patients and ward beds where they were admitted are indicated. Recipients 3R1 and 3R2 shared the same room, while recipient 4R1 was hospitalized three doors next to 3R1 and 3R2.

Supplementary fig 3.

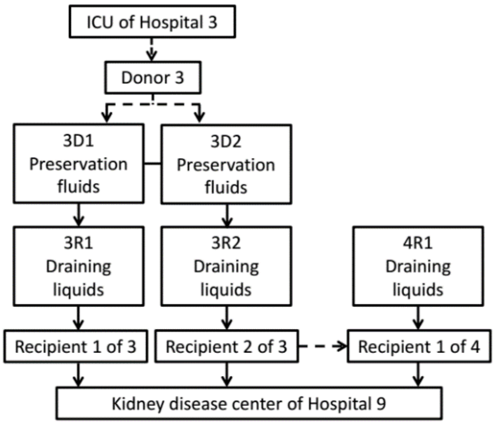

Fig S3. Putative map of A. baumannii transmission during the outbreak. The A.baumannii isolate of 3R1 and 3R2 was originally from the donor and was from the ICU where the donor stayed. 4R1 was transmitted from 3R1 and 3R2 via medical manipulation.
